# Associations between symptom change and treatment satisfaction in specialized eating disorder treatment

**DOI:** 10.1186/s40337-026-01657-z

**Published:** 2026-05-28

**Authors:** Rita Ueland, Øyvind Rø, Yngvild S. Danielsen

**Affiliations:** 1 Department of Clinical Psychology, University of Bergen, Bergen, Norway; 2 Regional Department for Eating Disorders, Division of Mental Health and Addiction, Oslo University Hospital, Oslo, Norway; 3 Regional Department of Eating Disorders, Haukeland University Hospital, Bergen, Norway; 4 Faculty of Medicine, Institute of Clinical Medicine, University of Oslo, Oslo, Norway

**Keywords:** Eating disorder, Treatment satisfaction, Clinical outcome

## Abstract

**Background:**

Patients receiving treatment for eating disorders often report dissatisfaction and negative treatment experiences, which may partly reflect the egosyntonic nature of these illnesses. This study aimed to examine different aspects of treatment satisfaction among patients receiving specialized eating disorder treatment, both inpatient and outpatient, and to investigate whether clinical outcomes were associated with treatment satisfaction.

**Methods:**

The sample consisted of 774 patients from Norway, adolecents and adults, all diagnosed with an ICD-10 eating disorder including anorexia nervosa (F50.0, F50.1), bulimia nervosa (F50.2, F50.3) and other more unspecified EDs (F50.4, F50.8, F50.9). Patient data was retrieved from the Norwegian Quality Register for Treatment of Eating Disorders. Hierarchical regression analyses were conducted to assess whether changes in eating disorder symptoms—measured by the Eating Disorder Examination Questionnaire (EDE-Q 6.0) and the Clinical Impairment Assessment (CIA 3.0)—as well as changes in body mass index (BMI) and general psychopathology—measured with the Symptom Checklist-Revised (SCL-90-R) and the Strengths and Difficulties Questionnaire (SDQ)—predicted scores on three different measures of treatment satisfaction: self-perceived treatment outcome, experience with treatment and total satisfaction.

**Results:**

Improvement in eating disorder related daily functioning was significantly and positively associated with all measures of treatment satisfaction and emerged as the strongest predictor. Improvements in eating disorder symptoms and BMI were associated with more positive evaluations of treatment outcome and total satisfaction, but not with patients’ experiences of treatment. Additionally, patients who received inpatient treatment reported significantly poorer treatment experiences than those treated in outpatient settings. Changes in general psychopathology were not associated with any satisfaction outcomes.

**Conclusions:**

Improvements in eating disorder related symptoms, daily functioning, and weight restoration were associated with higher treatment satisfaction, whereas improvements in general psychopathology were not. Findings indicated that patients may be satisfied with treatment in general despite the egosyntonic nature of the disease, and that treatment level may be of significance.

**Supplementary Information:**

The online version contains supplementary material available at 10.1186/s40337-026-01657-z.

## Abstract (plain language summary)

People receiving treatment for eating disorders often report varying levels of satisfaction with their care. This study examined changes in eating disorder symptoms, daily functioning, body weight, and general mental health, and how these changes were associated with different measures of treatment satisfaction: self-perceived treatment outcome, experience with treatment and total satisfaction. Results showed that changes in all measures, except general mental health, were associated with higher treatment satisfaction. For instance, improvements in daily functioning related to eating disorder symptoms were strongly associated with all aspects of treatment satisfaction and were the most important factor overall. Improvements in eating disorder related symptoms and body weight were linked to satisfaction with treatment outcomes and total satisfaction. However, patients receiving in-patient treatment reported more negative experiences during treatment.

**Supplementary Information:**

The online version contains supplementary material available at 10.1186/s40337-026-01657-z.

## Background

Eating disorders (EDs) are mental illnesses characterized by abnormal attitudes and behaviours related to food, eating, body shape and weight, such as anorexia nervosa (AN), bulimia nervosa (BN) and binge eating disorder (BED) [1]. EDs frequently co-occur with other mental disorders such as anxiety and depression [2] and are associated with somatic complications and elevated mortality [3, 4]. Patients in eating disorder treatment quite often report negative experiences from treatment [5–7] and premature termination of treatment is common [8]. Treatment is often perceived as overly focused on food and weight, with limited attention to the individual or the therapeutic relationship [9]. Restrictive, biomedical approaches may further marginalise patients’ identity, values, and lived experiences, with reports of reduced autonomy and being treated as part of a homogeneous diagnostic group [6, 10].

ED pathology has been characterized as partly egosyntonic in nature [11], indicating that the symptoms may be experienced as serving a protective function for identity. The ED symptoms often provide an experience of security, control, and a feeling of safety and comfort in parallel to other negative social and health complications [11]. As ED behaviors often immediately relieve or protect against psychological pain, individuals may value their ED and perceive it as adaptive [12], despite severe somatic consequences [2, 13], reduced quality of life [3, 14] and poorer life satisfaction [15]. Starting treatment would involve targeting the ED behaviors and symptoms, and would therefore mean letting go of any control, safety and comfort the ED may have provided. Treatment would also most often involve monitoring weight, and re-gaining lost weight for patients with underweight [16], and some individuals with EDs are also admitted to involuntary treatment. Specialized ED treatment is delivered on a spectrum of time and support, ranging from outpatient treatment sessions to day care and inpatient treatment. The treatment process most often entail high levels of anxiety and may lead to conflicting interests with health care professionals in addition to increased ambivalence towards recovery [11]. This variety of settings and challenges that patients may face during treatment [17] are likely to impact their treatment experience and reported treatment satisfaction [18].

Treatment satisfaction represents a patient’s evaluation of the care received relative to their expectations [19], and may encompass multiple dimensions of the treatment process, including interpersonal experiences with healthcare professionals, involvement in and individualisation of treatment, perceived symptom improvement and psychological well-being, as well as an overall appraisal of these elements. Knowledge about how patients both experience and evaluate treatment is highly valuable when delivering and developing healthcare services [19, 20]. Treatment experiences have been shown to influence both clinical outcome [21] and levels of satisfaction [6]. However, the literature is seemingly scarce in regard to how, or if, treatment outcome is associated with treatment satisfaction.

Follow-up studies of children and adolescents that have received treatment for eating disorders have not found a clear association between treatment satisfaction and clinical improvement [22, 23]. Results have shown patients to either be satisfied or dissatisfied independently of the clinical outcome, several years post treatment [22, 23]. However, these studies consisted of small sample sizes (*n* < 35) and patients diagnosed with AN only.

In contrast, a three-year follow-up study of an adult ED population (*n* = 469) compared satisfied with unsatisfied patients and found that less satisfied patients had higher levels of ED-symptoms [5]. Such findings may indicate a possible association between satisfaction and clinical outcome. However, it should be noted that the authors pointed out some psychometric limitations in regard to the satisfaction questionnaire, as it consisted of few questions with only a three-point scale. In addition, the study was conducted two decades ago. Measures of clinical outcome and levels of satisfaction, and their association, may therefore be different today as treatment settings and delivery have developed.

How change in weight might influence treatment satisfaction is also not widely studied. Weight gain has been demonstrated to significantly predict reduction in overall ED pathology in treatment of anorexia nervosa [24]. However, the focus on weight gain in treatment may also influence how patients experience treatment. In a qualitative study patients receiving inpatient treatment reported an experience of excessive focus on weight compromising psychological wellbeing and even treatment goals [6]. Another qualitative study revealed similar findings as patients expressed high degree of dissatisfaction with treatment, partly due to the perception of treatment being overly focused on weight [9].

There are some methodological shortcomings concerning previous research examining the association between clinical outcome and treatment satisfaction. Firstly, the sample sizes tend to be small [5, 22, 23]. Secondly, satisfaction has previously been assessed 18 months post treatment at the earliest [5, 22, 23]. Whether satisfaction is measured during treatment, right after treatment or at follow-up may influence how a patient perceives the treatment [6], thus affecting reported level of satisfaction.

The primary aim of this study was to investigate whether change in ED symptoms from pre- to post treatment could predict variance in treatment satisfaction. Secondary aims were to investigate whether an increase in body mass index, in patients with underweight at the start of treatment, would be associated with treatment satisfaction at the end of treatment. Further, if changes in general mental health symptoms during treatment could predict variance in treatment satisfaction. Another aim was to examine how age, gender, diagnosis, and treatment setting may influence treatment satisfaction.

## Methods

### Study design and setting

Anonymized data was collected from the Norwegian quality register for treatment of eating disorders (NorSpis), a national medical register. Patients registered between 2017 and 2023 were included in this study. All specialized psychiatric units conducting treatment for eating disorders in Norway are encouraged to report data to NorSpis using defined clinical measures and measures of satisfaction. Clinical symptoms are assessed at the start and at the end of treatment, whereas treatment satisfaction is only measured at the end of treatment. Until December 2023, registration was done manually either by the treatment provider or a mercantile at the units. Patient-reported measures contain ED symptomatology, general mental health symptoms and treatment satisfaction. Information reported by the treatment provider comprises diagnosis, weight and height, treatment level and treatment duration.

The application of data disclosure was approved 12.04.24 by the professional council of NorSpis.

In Norway most specialized ED treatment is delivered through the national public health system financed by the state, but a few private clinics also offer specialized treatment. Treatment may be delivered in outpatient, day-patient, or inpatient formats, provided within specialized hospital wards, general outpatient clinics, or dedicated specialist centres. Treatment units typically consist of multidisciplinary teams, including psychologists, psychiatrists, physicians and nurses. The national guidelines for treatment of EDs recommends Family-based treatment for eating disorders as a first choice in children and adolescents, while for adults the recommendation is psychotherapies targeting eating disorder symptoms, with cognitive behavioral treatment for EDs as a specified option [25].

### Participants

Data analyzed in the current study comprised a total of 774 patients, adolescents and adults, all diagnosed with an eating disorder and with completed assessment of satisfaction at the end of treatment. In this sample, treatment duration ranged from three to 1016 days for the inpatient treatment setting (*M* = 134.10, *SD* = 127.47), and eight to 1714 days for the outpatient treatment setting (*M* = 345.69, *SD* = 302.65).

It was decided to group patients into three main categories. The first category was named Anorexia Nervosa (AN) and included patients diagnosed with either F50.0 Anorexia Nervosa or F50.1 Atypical Anorexia Nervosa. In the second category, Bulimia Nervosa (BN), patients diagnosed with F50.2 Bulimia Nervosa and F50.3 Atypical Bulimia Nervosa were included. The third category included patients diagnosed with F50.4 Overeating associated with other psychological disturbances, F50.8 Other eating disorders or F50.9 Eating disorder unspecified, and was unitedly labeled Unspecified (US). All patients were diagnosed based on ICD-10 [26] by approved health care workers.

There was great variation in the type of treatment received. It was therefore decided to divide patients into two groups. One group with patients that had received inpatient treatment and one group with patients that had not (outpatient). Table 1 provides a description of baseline characteristics of the participants.

**Table 1:** Descriptives of patients at baseline (n = 774)

Variable	*N* (%)	Minimum	Maximum	*Mean*	*SD*
Age		13.0	71.0	26.47	9.62
Female	756 (97.7)				
Male	18 (2.3)				
BMI	766 (99.0)	10.4	63.0	20.81	7.79
BMI < 18.5	371 (47.9)	10.4	18.4	16.04	1.57
AN	511 (66.0)				
BN	150 (19.4)				
US	113 (14.6)				
Inpatient	380 (49.1)				
Outpatient	394 (50.9)				

BMI: Body Mass Index; AN: Anorexia Nervosa (F50.0 & F50.1); BN: Bulimia Nervosa (F50.2 & F50.3); US: Unspecified (F50.4 & F50.8 & F50.9)

### Measures

#### Treatment satisfaction

Three measures of treatment satisfaction were evaluated at the end of treatment: self-perceived treatment outcome, experience with treatment and total satisfaction. Patients answered a total of nine questions (Table 2). Low scores indicated treatment dissatisfaction and high scores indicated treatment satisfaction. The psychometric properties of these questionnaires have not been examined in previous literature [27]. However, the satisfaction questionnaires used in this study were developed in collaboration with the Norwegian Institute of Public Health based on items from previously validated surveys [28], for example the NORPEQ Patient Experiences Questionnaire, which shows good evidence of reliability and validity (Cronbach’s α = 0.85) [29].

**Table 2:** Measurements of treatment satisfaction

Question	Patient (*n* = 774)	
	*Mean*	*SD*
*Subscale experience*		
1. Did the clinicians talk to you in a way that was easy to understand?	3.62	0.58
2. Do you have confidence in the clinicians' professional skills?	3.54	0.70
3. Did you receive sufficient information about your diagnosis or medical condition(s)?	3.32	0.78
4. Did you perceive the treatment as adapted to your situation?	3.11	0.92
5. Were you involved in decisions regarding your treatment?	3.18	0.87
6. Did you perceive the treatment unit’s work as well organised?	3.16	0.83
*Subscale total satisfaction*		
7. Overall, was the help and treatment you received at the treatment unit satisfactory?	3.23	0.83
9. Overall, what benefit have you had from the care at the treatment unit?	2.90	0.93
*Self-perceived treatment outcome*		
*How do you perceive the outcome of received treatment?*	2.57	0.75

Minimum (1); Maximum (5); Low scores indicate dissatisfaction, high scores indicate satisfaction

Self-perceived treatment outcome is measured by asking patients, “How do you perceive the outcome of received treatment?” on a five-point scale. Low scores indicate worsening, and high scores indicate less or no ED symptoms anymore. Response categories were 1 (*deterioration*)*,* 2 (*no change*)*,* 3 (*some improvement*)*,* 4 (*distinct improvement*) *or 5* (*not an issue anymore*)*.*

Patient experience and total satisfaction was measured with The Patient Experiences in Specialist Health Care Questionnaire [27, 28]. The questionnaire consists of ten items with up to six response categories, and comprises four subscales: Experience, Total satisfaction, Availability and Safety. The subscales Availability and Safety were omitted from the final analysis on the basis of not being relevant for the current study. The subscale Experience consists of question one through six, and question seven and nine compose the subscale Total satisfaction. Response options for question one through seven: 1 (*not at all*), 2 (*to a small extent*)*,* 3 (*to some extent*)*,* 4 (*to a large extent*)*,* 5 (*to a very large extent*) or 6 (*not applicable*). Response options for question nine are 1 (*no benefit*)*,* 2 (*small benefit*)*,* 3 (*some benefit*)*,* 4 (*large benefit*)*,* 5 (*very large benefit*) *or* 6 (*not applicable*)*.* For each subscale, an average of the questions was converted to a scale ranging from 0 to 100. The sixth option (*not applicable*) was excluded from the subscales.

#### Body composition

Body Mass Index (BMI; kg/m^2^) is a measure of body composition [30]. BMI < 18.5 is defined as underweight in this study [31]. BMI was calculated by NorSpis using reported weight and height. Treatment providers measured weight and height by standard wall mounted measuring tapes and hospital scales at the start and end of treatment. BMI for children and youth was adjusted for gender and age [32, 33] to allow comparison with BMI in adults. Age and gender adjusted BMI were calculated by NorSpis using reference data from the Bergen Growth Study (BGS), as data from the BGS is assumed to be the most representative anthropometric reference data for Norwegian children and youths [34].

#### Measure of eating disorder symptoms

The Eating Disorder Examination Questionnaire 6.0 (EDE-Q) [35] is a self-report measure of ED psychopathology. The questionnaire obtains information from the last 28 days regarding core ED symptoms and behaviors. Patients are asked to estimate the frequency or actuality of 28 statements on a scale ranging from zero (*no days/not at all*) to six (*all days/very much*). EDE-Q consists of a global score and four subscales: dietary restraint, eating-, weight- and shape concern. The EDE-Q global score is calculated by averaging the four subscales. The EDE-Q has been shown to have good psychometric properties [36]. Also the Norwegian version used in this study has shown good internal consistency (*α* = 0.94) and test–retest reliability [37].

#### Measure of ED related psychosocial impairment

The Clinical Impairment Assessment Questionnaire 3.0 (CIA) [38] is a 16-item self-report measure of psychosocial difficulties due to the ED based on the past 28 days. Each item consists of four options, ranging from zero (*not at all*) to three (*a lot*), giving a maximum score of 48. CIA provides an understanding of how the ED affects daily functioning and consists of three subscales: personal impairment, social impairment and cognitive impairment. The questionnaire is found to have good psychometric properties, with high levels of internal consistency (*α* > 0.94) and test–retest reliability [39, 40].

#### Measures of general psychopathology

The Symptom Checklist Revised (SCL-90 R) for adults, and the Strengths and Difficulties Questionnaire (SDQ) for individuals under the age of 18, were included to investigate potential differences between improvement in general psychological symptoms and improvement in ED symptoms.

The SCL-90 R is a self-assessed questionnaire that aims to measure general psychological symptom load, and how the adult patient perceives different dimensions of their mental health [41]. SCL-90 R consists of 90 statements, which constitutes ten subscales: somatization, compulsive tendencies, interpersonal hypersensitivity, depression, anxiety, hostility, phobic anxiety, paranoid ideation, psychoticism. The Global Severity Index (GSI) is calculated by the overall average. GSI was the only scale from the SCL-90 R included in this study. The Norwegian version of SCL-90 R has shown adequate psychometric properties, with Cronbach's Alpha being above 0.95 for the global score [42].

The SDQ is a 25-item measure of behaviours and psychopathology in children and youth [43]. The questionnaire consists of five subscales with five items each; emotional symptoms, conduct problems, hyperactivity/inattention, peer relationship problems and prosocial behaviour. Subscale one through four generates the Total Difficulties Score, ranging from 0 to 40, used in the current study. Higher score indicates greater difficulties, where a total score above 17 is considered the cut-off for clinical impact [44]. The SDQ has been shown to have acceptable psychometric properties regarding internal consistency [45], concurrent validity [46] and test–retest reliability [47]. In a Norwegian sample Cronbach's alpha was 0.78 [48].

### Statistics

All analyses were performed in IBM SPSS Statistics (Version 29.0.2.0). Sample sizes vary for the different measures due to the number of patients with BMI under 18 and because of age grouping in the general measures. Global scores were used for EDE-Q, CIA, SCL-90-R and SDQ. Change scores were calculated by subtracting the start score from the end score (See Table 3). Negative values indicated a decrease in symptoms and an increase in weight. Preliminary analyses were conducted to ensure no violation of assumptions. Correlation analyses were conducted for all variables. A series of hierarchical multiple regression analyses was conducted to assess the ability of symptom change to predict levels of treatment satisfaction after controlling for the influence of age, gender, diagnosis and treatment level. Separate analyses were conducted for each of the three satisfaction variables, introducing predictors in a three-step hierarchical approach. For all models, age and gender were entered at Step 1, whereas diagnosis and treatment level were entered at Step 2. Change scores from clinical measures were entered at Step 3. Analyses regarding change in BMI only included patients registered as underweight at the start of treatment.

**Table 3:** Descriptives of clinical measures and change scores

Variable	Pre-treatment		Post-treatment		Change score			
	*Mean*	*SD*	*Mean*	*SD*	*Mean*	*SD*	*N*	*d*
EDE-Q	3.94	1.20	2.77	1.53	− 1.17	1.28	696	− 0.91
CIA	33.99	9.39	23.45	12.96	− 10.44	11.20	679	− 0.93
SCL-90	1.57	0.69	1.19	0.77	− 0.38	0.56	575	− 0.68
SDQ	15.87	5.82	14.00	5.53	− 1.59	4.27	84	− 0.37
BMI < 18.5	16.04	1.57	18.36	2.13	− 2.32	2.01	364	− 1.15

BMI: Body Mass Index; EDE-Q: Eating Disorder Examination Questionnaire 6.0; CIA: Clinical Impairment Questionnaire; SCL-90 R: Symptom Checklist Revised; SDQ: Strengths and Difficulties Questionnaire; N: number of patients with completed satisfaction measures in addition to clinical measures; d: Cohen´s d

## Results

Treatment satisfaction for the total patient population in this study was 3.18 on a scale from 1 to 5 (mean average of all items, see Table 2). Ranging from a mean of 3.2 on the treatment experience subscale to 3.06 on the total satisfaction subscale, and 2.57 on self-perceived treatment outcome. Results also showed an overall reduction in both ED related symptoms and general measures of symptomatology post treatment (see Table 3). Descriptive statistics (see Tables 1 and 3) and correlation analysis (see Supplementary Material) were performed prior to the hierarchical regression analyses.

### Change in ED symptoms as a predictor of treatment satisfaction

Results from the hierarchical regression analysis exploring the impact of reduction in ED pathology measured by EDE-Q, and increased everyday functioning measured by CIA, on the three variables of treatment satisfaction are presented in Table 4.

**Table 4:** Hierarchical regression summary predicting treatment satisfaction with EDE-Q and CIA

Step and predictor variable	Self-perceived treatment outcome			Patient experience			Total satisfaction		
	β	*R*^*2*^	*Δ R*^*2*^	β	*R*^*2*^	*Δ R*^*2*^	β	*R*^*2*^	*Δ R*^*2*^
Step 1		0.00	0.00		0.00	0.00		0.01	0.01**
Age	0.01			0.04			0.11**		
Gender	0.05			0.03			0.05		
Step 2		0.01	0.01*		0.05	0.04***		0.03	0.01**
Age	− 0.01			− 0.01			0.07		
Gender	0.05			0.03			0.05		
Diagnosis	0.04			0.08			0.10*		
Treatment level	− 0.08			-0.17			− 0.05		
Step 3		0.28	0.27***		0.12	0.07***		0.18	0.15***
Age	0.04			0.01			0.12**		
Gender	0.04			0.03			0.04		
Diagnosis	0.00			0.07			0.07		
Treatment level	− 0.01			− 0.14***			0.00		
EDE-Q	− 0.19***			− 0.10			− 0.16**		
CIA	− 0.36***			− 0.19***			− 0.26***		

EDE-Q: Eating Disorder Examination Questionnaire 6.0; CIA: Clinical Impairment Questionnaire; β: standardized coefficients Beta

^*^p < 0.05

**p < 0.01

***p < 0.001

#### Self-perceived treatment outcome

The total variance explained by the model as a whole was 28%, F (6, 672) = 44.19, *p* < 0.001. EDE-Q and CIA explained 27% of this variance after controlling for age, gender, diagnosis and treatment level (*F* change (2, 672) = 126.74, *p* < 0.001). In the final model, only the clinical measures emerged as statistically significant predictors, with CIA recording a higher semipartial correlation value (*sr* = − 0.23, *p* < 0.001) than EDE-Q (*sr* = − 0.12, *p* < 0.001). Improvement in symptoms was associated with higher levels of satisfaction related to self-perceived treatment outcome for both measures.

#### Patient experience

The total variance explained by the model as a whole was 12% (*F* (6, 672) = 15.31, *p* < 0.001). EDE-Q and CIA explained 7% of this variance (*F* change (2, 672) = 27.89, *p* < 0.001). In the final model, treatment level (*sr* = − 0.13, *p* < 0.001) and CIA (*sr* = − 0.12, *p* < 0.001) emerged as the only statistically significant predictors. This indicates that both outpatient treatment and improvement in the CIA was associated with higher levels of satisfaction related to patient experiences of treatment.

#### Total satisfaction

The total variance explained by the model as a whole was 18% (*F* (6, 672) = 24.54, *p* < 0.001). EDE-Q and CIA explained 15% of this variance (*F* change (2, 672) = 61.61, *p* < 0.001). Age emerged as a significant contributor in addition to the clinical measures in the final model. CIA recorded a higher semipartial correlation (*sr* = − 0.16, *p* < 0.001) than EDE-Q (*sr* = − 0.10, *p* = 0.004) and age (*sr* = − 0.11, *p* = 0.002). Higher age and improvement in both CIA and EDE-Q were associated with higher levels of satisfaction.

### Change in BMI for underweight patients as predictor of treatment satisfaction

Results from the hierarchical regression analysis conducted to explore the impact of change in BMI for underweight patients on three variables of treatment satisfaction are presented in Table 5.

**Table 5:** Hierarchical regression summary, BMI < 18.5 predicting Treatment Satisfaction

Step and predictor variable	Self-perceived treatment outcome			Patient experience			Total satisfaction		
	*β*	*R*^*2*^	*Δ R*^*2*^	*β*	*R*^*2*^	*Δ R*^*2*^	*β*	*R*^*2*^	*Δ R*^*2*^
Step 1		0.00	0.00		0.00	0.00		0.01	0.01
Age	− 0.05			0.01			0.08		
Gender	0.04			0.03			0.08		
Step 2		0.00	0.00		0.01	0.01		0.01	0.00
Age	− 0.06			0.01			0.08		
Gender	0.04			0.05			0.07		
Diagnosis	0.04			− 0.04			0.04		
Treatment level	− 0.00			− 0.12*			0.02		
Step 3		0.29	0.29***		0.10	0.09***		0.22	0.21***
Age	0.01			0.05			0.14**		
Gender	− 0.01			0.03			0.03		
Diagnosis	0.07			− 0.02			0.07		
Treatment level	− 0.01			− 0.12*			− 0.00		
EDE-Q	− 0.20**			− 0.09			− 0.16*		
CIA	− 0.26***			− 0.17*			− 0.17*		
BMI	− 0.22***			− 0.11*			− 0.26***		

BMI: Body Mass Index; EDE-Q: Eating Disorder Examination Questionnaire 60.0; CIA: Clinical Impairment Questionnaire; β: standardized coefficients Beta

^*^p < 0.05

**p < 0.01

***p < 0.001

#### Self-perceived treatment outcome

The total variance explained by the model as a whole was 29% (*F* (7, 305) = 18.11, *p* < 0.001). BMI, EDE-Q and CIA explained 28% of this variance after controlling for age, gender, diagnosis and treatment level (*F* change (3, 305) = 41.29, *p* < 0.001). In the final model, the three clinical measures were the only statistically significant predictors, with change in BMI recording a higher semi partial correlation value (*sr* = − 0.20, *p* < 0.001) than EDE-Q (*sr* = − 0.13, *p* = 0.007) and CIA (*sr* = − 0.16, *p* < 0.001). This indicates that an increase in BMI and a decrease in symptoms were associated with higher levels of satisfaction related to self-perceived treatment outcome.

#### Patient experience

The total variance explained by the model as a whole was 10% (*F* (7, 305) = 5.06, *p* < 0.001). The last step explained 9% of this variance (*F* change (3, 305) = 9.95, *p* < 0.001). In the final model, change in BMI was not significant (*sr* = − 0.10, *p* = 0.053). However, treatment level (*sr* = − 0.11, *p* = 0.034) and CIA (*sr* = − 0.11, *p* = 0.045) were statistically significant. This indicates that both outpatient treatment and lower score on CIA was associated with higher levels of satisfaction related to patient experiences of treatment.

#### Total satisfaction

The total variance explained by the model as a whole was 22% (*F* (7, 305) = 12.55, *p* < 0.001). The final step explained 21% of this variance (*F* change (3, 305) = 27.47, *p* < 0.001). In the final model, age and all clinical measures were significant, with change in BMI recording a higher semi partial correlation value (*sr* = − 0.24, *p* < 0.001) than age (*sr* = 0.14, *p* = 0.005), EDE-Q (*sr* = − 0.11, *p* = 0.036) and CIA (*sr* = − 0.11, *p* = 0.030). This indicates that higher age, an increase in BMI and a decrease in symptoms were associated with higher levels of satisfaction.

### Change in SCL-90 R as predictor of treatment satisfaction

Hierarchical regression analysis was conducted to explore the impact of improvement in general psychological symptoms on treatment satisfaction. SCL-90 R, EDE-Q and CIA were added in the third and final step.

#### Self-perceived treatment outcome

The total variance explained by the model as a whole was 30% (*R* square = 0.30, *F* (7, 531) = 33.34, *p* < 0.001). SCL-90 R together with EDE-Q and CIA explained an additional 28% of the variance after controlling for age, gender, diagnosis and treatment level (*R* squared change = 0.28, *F* change (3, 531) = 72.62, *p* < 0.001). However, SCL-90 R was not a statistically significant predictor (β = -0.05, *sr* = − 0.04, *p* = 0.285).

#### Patient experience

The total variance explained by the model as a whole was 14% (*R* square = 0.14, *F* (7, 531) = 12.55, *p* < 0.001). The third step explained an additional 9% of the variance (*R* squared change = 0.09, *F* change (7, 531) = 18.02, *p* < 0.001), however, SCL-90 R was not a significant contributor (β = − 0.005, *sr* = − 0.003, *p* = 0.935).

#### Total satisfaction

The total variance explained by the model as a whole was 19% (*R* square = 0.19, *F* (7, 531) = 17.52, *p* < 0.001). The final step explained an additional 16% of the variance (*R* squared change = 0.16, *F* change (3, 531) = 34.26, *p* < 0.001), however, SCL-90 R was not a significant contributor (β = − 0.04, *sr* = − 0.03, *p* = 0.501).

### Change in SDQ as predictor of treatment satisfaction

Hierarchical regression analysis was conducted to explore the impact of improvement in general psychological symptoms on treatment satisfaction. SDQ, EDE-Q and CIA were added in the third and final step.

#### Self-perceived treatment outcome

The total variance explained by the model as a whole was 19% (*R* square = 0.19, *F* (7, 70) = 2.4, *p* = 0.029). SDQ together with EDE-Q and CIA explained an additional 18% of the variance after controlling for age, gender, diagnosis and treatment level (*R* squared change = 0.18, *F* change (3, 70) = 5.24, *p* = 0.003). SDQ was not a significant contributor (β = − 0.01, *sr* = 0.01, *p* = 917).

#### Patient experience

The total variance explained by the model as a whole was 13%, however the model was not statistically significant (*R* square = 0.13, *F* (7, 70) = 1.47, *p* = 0.191). The third step explained an additional 5% of the variance, however, this was not a significant contribution (*R* squared change = 0.05, *F* change (3, 70) = 1.2, *p* = 0.304). SDQ was not a significant contributor (β = − 0.26, *sr* = − 0.20, *p* = 0.72).

#### Total satisfaction

The total variance explained by the model as a whole was 19% (*R* square = 0.19, *F* (7, 70) = 2.4, *p* = 0.029). The final step explained an additional 10% of the variance (*R* squared change = 0.10, *F* change (3, 70) = 2.8, *p* = 0.045), however, SDQ was not a significant contributor (β = − 0.11, *sr* = − 0.09, *p* = 0.419).

## Discussion

The aim of this study was to explore the association between clinical outcome measures and patients’ satisfaction with treatment for eating disorders. Treatment satisfaction consisted of three variables: self-perceived treatment outcome, patient experience and total satisfaction. Self-perceived treatment outcome is how the patients subjectively perceive health status post treatment, while patient experiences refer to satisfaction with the structure of treatment and alliance with health care providers. Total satisfaction is defined as patients’ overall satisfaction with their treatment and benefit of the received care.

Results showed a significant association between improvement in ED related impairment and higher level of all three measures of treatment satisfaction. Reduction in eating disorder symptoms was related to better self-perceived treatment outcome and higher score on total satisfaction, but not with patient experience. In addition, inpatient treatment was associated with more negative patient experiences, and higher age was associated with higher scores on total satisfaction. For patients that were underweight at the start of treatment, an increase in BMI during treatment was associated with better self-perceived treatment outcome and total satisfaction, but not with patient experiences from treatment. Changes in general psychopathology were not associated with any of the treatment satisfaction outcomes.

### Change in ED symptoms and impairment as predictor of treatment satisfaction

Improvement in ED specific symptoms (EDE-Q) and increased everyday functioning (CIA) had the largest contribution to variance in self-perceived treatment outcome and the least in patient experience. Results illustrate that patients with symptom improvement may be satisfied with treatment outcomes, and at the same time they might be dissatisfied with the organization and delivery of the treatment. A possible interpretation could be that the patients value the improvement in function and ED-symptoms despite experiencing the treatment as quite demanding. Rankin et al.’s meta of qualitative studies on patient experiences from ED treatment highlight that some patients might value the importance of some treatment interventions post treatment, despite having had quite negative experiences during treatment [6]. Research has also found that the value of improved everyday functioning has the ability to exceed the need for total absence of symptoms [49, 50]. Such findings are interesting as our results showed that change in CIA overall contributed more to the variance in satisfaction, than change in EDE-Q.

In our results, treatment level emerged as a modifying factor and inpatient treatment was associated with poorer patient experiences. In general inpatient treatment is found to be less favourable than outpatient treatment, and only used when outpatient treatment is deemed unsafe or that more intensive support is needed [51]. Against this backdrop, it is not surprising that there is a difference in the experience of inpatient versus outpatient treatment. Complying with a strict set of rules 24 h a day rather than coming to sessions a couple of hours a week constitutes two quite different settings. For instance, patients have reported that inpatient treatment is experienced as surrendering both autonomy and the right to decide your own structure of everyday life [6], whereas outpatient treatment may maintain their autonomy to a greater extent. When examining the reasons why patients are less satisfied with inpatient treatment, dissatisfaction has been linked to the organization and structure of treatment [52], which are some of the aspects also measured in the current study. For example, patients have reported inpatient treatment to be insufficiently individualized and overly focused on standardized protocols for physical restoration, leaving them with negative experiences of not being seen as a whole person, in turn influencing treatment satisfaction [6].

### Change in BMI for underweight patients as predictor of treatment satisfaction

Results showed an association between increased weight and two of the treatment satisfaction measures, better self-perceived treatment outcomes and total satisfaction. This finding indicates that weight gain is experienced as related to recovery by most patients at the end of treatment, even though weight gain in previous studies also is found to be experienced as contradictory to the egosyntonic nature of the illness [11], and often described as a fear inducing treatment element [53]. A possible interpretation may be that patients understand the necessity of weight gain towards recovery, simultaneously as they may be dissatisfied with the framework for weight regain and the process itself, as well as the experienced hardship of dealing with the anxiety that arises when gaining weight.

For the total satisfaction subscale, higher age was associated with higher levels of satisfaction both for change in ED-symptoms and BMI. This is in contrast to findings from a previous study on adolescents in eating disorder treatment [54]. The majority of our sample, however, consisted of adult patients suggesting there might be differences in satisfaction regarding treatment for adults compared to adolescents. An adult patient may experience higher levels of self-determination and autonomy regarding initiation and completion of treatment. In contrast, adolescents often undergo treatment with their parents as the main agents of change [55] during a developmental phase where increased detachment from parents is normative [56]. Therefore, the difference in findings may not be as surprising after all.

### Change in general psychological symptoms as predictor of treatment satisfaction

Psychiatric co-morbidity is common with eating disorders, with more than half of the population having anxiety and mood disorders and nearly a third reporting post-traumatic stress [2]. Psychiatric co-morbidity is often considered an obstacle to effectiveness of ED treatment and general psychological symptom load has previously been found to be negatively correlated with treatment satisfaction [57]. However, change in general psychological symptoms did not significantly predict treatment satisfaction in our sample. This could indicate that change in ED symptoms and improvement of daily function is experienced as most important for recovery in this group, independent of persistence or improvement in other psychological symptoms. Also, participants in this study were treated at specialized ED centers due to the high severity and burden of their symptoms. Because the treatment they received specifically targeted the eating disorder, it is natural that their treatment satisfaction ratings primarily reflect changes in ED-related symptoms. However, given that ​​change in general psychological symptoms was not significantly associated with treatment satisfaction in the present study, future research could consider examining the association between treatment satisfaction and more general measures of quality of life or functioning.

### Strengths and limitations

A strength to this study is the large sample size, compared to previous research [5, 22, 23]. This study also has a broader inclusion of ED diagnoses in contrast to studies that only included participants diagnosed with AN [22], even though the majority of patients in our sample were also diagnosed with AN (66%) suggesting results may be most representative for this specific subgroup. Another strength to this study is the measurement of treatment satisfaction at the end of treatment. This may have hindered other factors to influence patient evaluation, compared to measurement at a later point in time. Conversely, Solberg et al. argue that measuring satisfaction at the end of treatment may lead to transient findings, and that a later follow-up may prevent this [58]. It would have been interesting to include a follow-up measure, as Solberg et al. [58] did not find a significant association between clinical improvement and satisfaction in their study. However, this was not possible in our analyses as NorSpis does not obtain such long-term data, which represents an important limitation to this study. It should be acknowledged that inpatient and outpatient treatment were analysed as broad categories in this study. As both settings may encompass diverse treatment approaches and levels of intensity, this may have introduced unaccounted within-group variability and potentially influenced the observed associations. Another limitation to this study is that the psychometric properties of the treatment satisfaction measures have not been sufficiently investigated [27, 28]. We were not able to calculate Cronbach's Alpha for the different measures included in our study as the dataset extracted from NorSpis did not include item variables.

### Implications

Results show that improvement in ED symptoms and weight only accounts for a part of treatment satisfaction, indicating the presence of other factors influencing treatment satisfaction. Further investigation of such factors could provide a better basis for clinical implications.

Results also highlight that improved everyday functioning, as a result of reduction in ED symptoms, is important for treatment satisfaction and therefore an important focus in treatment. Results also underline the importance of weight gain as a treatment aim for patients with underweight, as increased BMI seemed to be of importance for satisfaction with treatment outcome, despite the egosyntonic nature of symptoms.

Our results demonstrated lower satisfaction with the organization of inpatient treatment, despite symptom improvement. This is not surprising due to the inherent difference of an out-patient versus inpatient setting but anyhow underscores the need for optimal routines for user involvement, especially in the inpatient setting. In line with this, a growing body of research highlights the value of holistic and multidisciplinary approaches in eating disorder treatment. Such approaches emphasise person-centred and biopsychosocial care [59], where balancing structure with autonomy may improve both satisfaction and outcomes [5, 10]. Qualitative findings further suggest that enhancing inpatient experiences may depend on strengthening therapeutic relationships and supporting patients in being understood as more than their illness [6, 10, 59] including fostering a sense of identity beyond the eating disorder [60].

## Conclusion

Improvement in ED symptoms and increased BMI when being underweight was found to be associated with treatment satisfaction. In particular, regaining everyday functioning seemed to be of importance for satisfaction. However, treatment level and age were also found to be predicting factors. Results highlight the complexity of examining user satisfaction as the included variables only accounted for up to 30% of the variance in treatment satisfaction.

## Supplementary Information

Supplementary Material 1.

## Data Availability

The raw/processed data required to reproduce the above findings cannot be shared at this time due to legal/ ethical reasons but could be made available upon request for control purposes until the ethical approval requires them deleted.

## Abbreviations

ED: Eating disorder
AN: Anorexia nervosa
BN: Bulimia nervosa
BED: Binge eating disorder
NorSpis: Norwegian quality register for treatment of eating disorders
US: Unspecified
BMI: Body mass index
BGS: Bergen Growth Study
EDE-Q: Eating Disorder Examination Questionnaire
CIA: Clinical Impairment Assessment Questionnaire
SCL-90 R: Symptom Checklist Revised
SDQ: Strengths and Difficulties Questionnaire
GSI: The Global Severity Index
