# Supplementary material for: Associations between symptom change and treatment satisfaction in specialized eating disorder treatment
Source: J Eat Disord. 2026 May 28;14:173. doi: 10.1186/s40337-026-01657-z (PMC13411307; doi:10.1186/s40337-026-01657-z)
Supplement: Supplementary file 1 — Supplementary Material 1. [file 40337_2026_1657_MOESM1_ESM.docx]

| **Table 1**  *Descriptives and Pearson Correlations for clinical outcome measures and treatment satisfaction* | | | | | | | | | |
| --- | --- | --- | --- | --- | --- | --- | --- | --- | --- |
| Variable | *n* | *M* | *SD* | 1 | 2 | 3 | 4 | 5 | 6 |
| 1.EDE-Q | 696 | -1.17 | 1.28 | - |  |  |  |  |  |
| 2.CIA | 697 | -10.44 | 11.20 | .766** | - |  |  |  |  |
| 3.BMI | 755 | -1.37 | 2.19 | .064 | .150** | - |  |  |  |
| 4.Self-Perceived Treatment Outcome | 774 | 3.43 | .751 | -.475** | -.512** | -.102** | - |  |  |
| 5.Patient Experience | 774 | 83.07 | 14.81 | -.267** | -.290** | .021 | .371** | - |  |
| 6.Total Satisfaction | 774 | 76.59 | 19.98 | -.361** | -.377** | -.081* | .620** | .678** | - |
| *Note:* *BMI, Body Mass Index; EDE-Q, Eating Disorder Examination Questionnaire 6.0; CIA, Clinical Impairment Questionnaire*  *Change score is reported for EDE-Q, CIA and BMI*  **p<.05 **p*<.001 | | | | | | | | | |

| **Table 2**  *Descriptives and Pearson Correlations for general measures of psychopathology and treatment satisfaction* | | | | | | | | |
| --- | --- | --- | --- | --- | --- | --- | --- | --- |
| Variable | *n* | *M* | *SD* | 1 | 2 | 3 | 4 | 5 |
| 1.SDQ | 84 | -1.59 | 4.27 | - |  |  |  |  |
| 2.SCL-90R | 575 | -.38 | .56 | - | - |  |  |  |
| 3.Self-Perceived Treatment Outcome | 774 | 3.43 | .751 | .234* | .394** | - |  |  |
| 4.Patient Experience | 774 | 83.07 | 14.81 | -.254* | -.229** | -.371** | - |  |
| 5.Total Satisfaction | 774 | 76.59 | 19.98 | -.302** | -.295** | -.620** | .678 | - |
| *Note:* *SCL-90 R, Symptom Checklist Revised; SDQ, Strengths and Difficulties Questionnaire*  *Change score is reported for SDQ and SCL-90R*  **p<.05 **p*<.001 | | | | | | | | |

| **Table 3**  *Descriptives and Spearman’s Correlations for control variables and treatment satisfaction* | | | | | | | | | | |
| --- | --- | --- | --- | --- | --- | --- | --- | --- | --- | --- |
| Variable | *n* | *M* | *SD* | 1 | 2 | 3 | 4 | 5 | 6 | 7 |
| 1. Age | 774 | 26.47 | 9.62 | - |  |  |  |  |  |  |
| 2.Gender^a^ | 774 |  |  | .021 | - |  |  |  |  |  |
| 3.Diagnosis^b^ | 774 |  |  | .298** | .073* | - |  |  |  |  |
| 4.Treatment Level^c^ | 774 |  |  | -.179** | .037 | -.364** | - |  |  |  |
| 5.Self-Perceived Treatment Outcome | 774 | 3.43 | .751 | -.039 | .050 | .092* | -.126** | - |  |  |
| 6.Patient Experience | 774 | 83.07 | 14.81 | .037 | .032 | .162** | -.212** | .368** | - |  |
| 7.Total Satisfaction | 774 | 76.59 | 19.98 | .106** | .057 | .132** | -.108** | .593** | .647** | - |
| *Note*:  *^a^ Female 0, Male 1*  *^b^ AN 1, BN 2, US 3*  *^c^ Outpatient 1, Inpatient 2*  **p*<.05 ***p*<.001 | | | | | | | | | | |
